# The oxygen‐sensing FixLJ represses nitrogen fixation in *Rhodopseudomonas palustris* in response to oxygen

**DOI:** 10.1002/mlf2.70067

**Published:** 2026-04-09

**Authors:** Lingwei Cui, Yan Zeng, Mengmei Wang, Lu Huang, Zheyi Wang, Ying Liu, Yanning Zheng

**Affiliations:** ^1^ State Key Laboratory of Microbial Diversity and innovative Utilization, Institute of Microbiology Chinese Academy of Sciences Beijing China; ^2^ College of Life Sciences University of Chinese Academy of Sciences Beijing China

**Keywords:** FixLJ system, nitrogenase, oxygen sensing, photosynthetic diazotroph, *Rhodopseudomonas palustris*

## Abstract

Biological nitrogen fixation in symbiotic diazotrophs is subject to oxygen regulation by an oxygen‐sensing FixLJ two‐component system under micro‐oxic conditions. However, it remains unclear whether this mechanism is conserved in free‐living diazotrophs. In this study, we discovered for the first time that FixLJ strongly inhibits the expression of *nifHDK* genes that encode molybdenum nitrogenase in response to oxygen. The deletion of *fixLJ* genes, whose expression was stimulated by oxygen, allowed a free‐living photosynthetic diazotroph *Rhodopseudomonas palustris* to express active nitrogenase and grow diazotrophically even under oxic conditions. The unphosphorylated FixJ protein showed high‐affinity binding to the promoter of nitrogenase gene cluster (P_*nifH*_) and strongly repressed the nitrogenase expression in response to oxygen. The transcriptional repression of *nifHDK* by FixJ reveals a new regulatory role for the FixLJ system. In addition, transcriptome analysis suggested that the FixLJ regulatory system also plays a role in the energy metabolism of *R. palustris*, probably through FixK regulation. This newly identified mechanism is speculated to allow *R. palustris* to rapidly shut down the synthesis of nitrogenase when exposed to oxygen, avoiding the build‐up of nitrogenase with impaired activity due to the lack of protection from oxygen damage.

## Impact statement

Nitrogenase, as an oxygen‐sensitive metalloenzyme, requires protection from oxygen damage. In symbiotic diazotrophs, the microoxia‐activated *cbb*_3_ terminal oxidase, regulated by the FixLJ system, establishes an anoxic microenvironment for nitrogen fixation. However, it has been found that free‐living diazotrophs fix nitrogen anoxically; oxygen protection is likely unnecessary. This study identified a novel function of the FixLJ system in a free‐living phototrophic diazotroph *Rhodopseudomonas palustris*. Under oxic conditions, FixJ directly represses nitrogenase expression, decreasing the cell's energy and nutrient consumption for survival.

## INTRODUCTION

The enzyme nitrogenase found in some bacteria and archaea is responsible for about half the conversion of nitrogen gas (N_2_) into biologically accessible ammonia (NH_3_) on the Earth, with the balance provided by the industrial Haber–Bosch process. Biological nitrogen fixation requires large amounts of ATP, and nitrogenase is an oxygen‐sensitive enzyme. For these reasons, nitrogenase is tightly regulated and synthesized by microbes only when it is needed and only under conditions where it will be active.

In *Bradyrhizobium japonicum*, the nitrogen‐fixing symbiont of soybeans, and in *Sinorhizobium meliloti*, the nitrogen‐fixing symbiont of alfalfa, a two‐component signal transduction system called FixLJ controls the expression of nitrogenase under micro‐oxic conditions^1^. The oxygen sensor FixL has a heme‐containing Per‐Arnt‐Sim (PAS) domain and a histidine kinase module. When oxygen is unavailable, the Asp residue of the response regulator FixJ is trans‐phosphorylated by autophosphorylated FixL in an extremely unstable manner with lower frequency^2^, ^3^. In *S. meliloti*, phosphorylated FixJ stimulates the transcription of *nifA*, which encodes a transcriptional activator of *nif* genes for nitrogenase synthesis^4^, ^5^. In addition, phosphorylated FixJ activates expression of the global transcriptional regulatory *fixK* gene^6^, ^7^. FixK, which is insensitive to oxygen, binds to a conserved palindromic operator sequence (TTGA‐N_6_‐TCAA) to regulate the expression of approximately 200 target genes in diverse α‐proteobacteria. In *B. japonicum*, FixJ activates the transcription of the *fixK₂* gene, which encodes a regulatory protein that further activates multiple genes such as *fixNOQP* and *fixGHIS*, essential for micro‐oxic, anoxic, or symbiotic growth^8^, ^9^. In *S. meliloti*, the FixJ protein functions as a regulator of the symbiotic response. FixJ activates the transcription of a wide range of genes essential for survival within the nodule, including those involved in micro‐oxic respiration (*ccoNOQP*), denitrification (*nap*, *nor*, and *nos* gene clusters), amino acid metabolism (*arcA1BC*, *SMa0680*, *SMa0682*, and *proB2*), stress response (*hspC2*, *SMa1147*, and *SMa1158*), and nodulation infection (*syrA*, *nodL*, and *noeA*)^10^. In *Rhodopseudomonas palustris*, the FixLJ–FixK system acts as a global regulator. FixK, which is activated by FixJ, primarily regulates genes involved in micro‐oxic respiration (*cco* and *hup*), synthesis of the photosynthetic apparatus, and phototrophic iron oxidation (*pioABC*)^11^, ^12^. In *Azorhizobium caulinodans*, FixK activates the transcription of *nifA*, suggesting that the FixLJ system can also indirectly control nitrogenase expression via the regulator FixK^13^.

The FixLJ oxygen‐sensing system has been well studied in symbiotic diazotrophs^14^, where it is clear that it guarantees that rhizobia can precisely balance the ATP generation by oxic respiration with oxygen‐sensitive nitrogen fixation. In this study, we investigated the role of FixLJ in a free‐living phototrophic diazotroph *R. palustris*, which is an excellent model to study nitrogenase. It encodes three forms of nitrogenase with three different transition metals at their active sites. In contrast to the rhizobia that make ATP by oxidative phosphorylation under oxic conditions, *R. palustris* generates the large amount of ATP required for nitrogenase activity by cyclic photophosphorylation under anoxic conditions^15^. *R*. *palustris* encodes FixLJ and FixK, and the activity of FixK was studied in some detail and found to regulate diverse functions, including micro‐oxic growth and photosynthesis^11^. *R. palustris fixJ* and *fixK* deletion mutants were tested for defects in growth under nitrogen‐fixing conditions and no defects were found^11^. From this, the authors concluded that the FixLJ–FixK system likely did not control nitrogen fixation. The relationships among FixLJ, FixK, and NifA remain unclear.

Here, we show that the expression of *fixLJ* is stimulated by oxygen and that unphosphorylated FixJ represses the expression of *R. palustris* nitrogenase by binding to the promoter region of the nitrogenase structural genes *nifHDK*. This represents a new strategy for regulation of nitrogenase gene expression—one that is active in response to oxygen.

## RESULTS

### The presence of the FixLJ two‐component system in *R. palustris*

**Figure 1:**
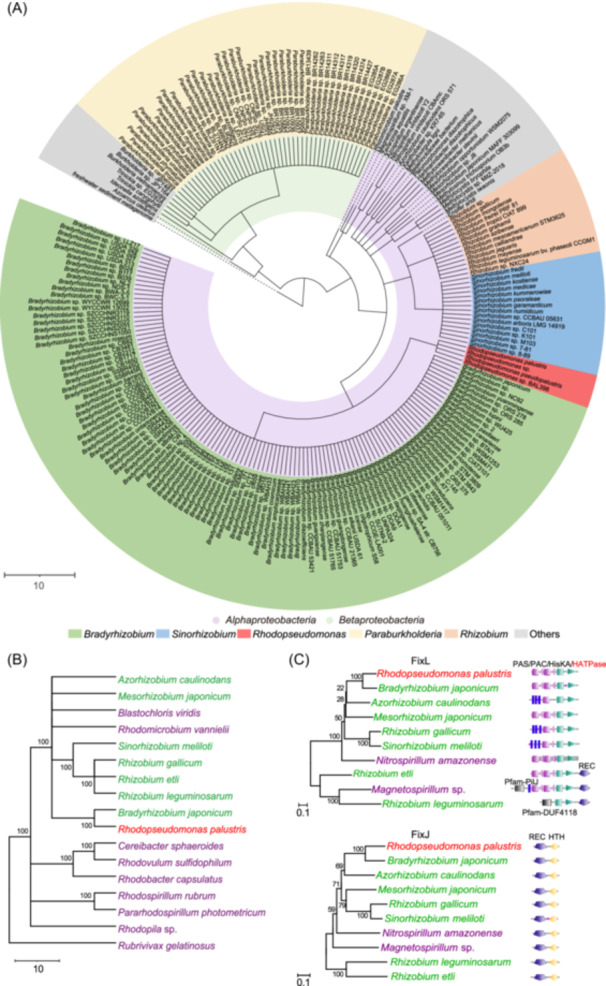
Bioinformatic analysis of FixL and FixJ. (A) Phylogenetic tree of the diazotrophs that have FixLJ proteins. The phylo genetic tree was generated using the PHYLIP tree file obtained from the common taxonomy tree in the NCBI database. The diazotrophic *Alphaproteobacteria* are highlighted in purple, while diazotrophic *Betaproteobacteria* are shown in light green (inner ring). *Rhodopseudomonas* strains are highlighted in red (outer ring). (B) Phylogenetic analysis based on the genomic DNAs of free‐living diazotrophs (shown in red or purple) and symbiotic diazotrophs (shown in green). (C) Phylogenetic tree and protein domain architecture of FixL and FixJ. The sequences from free‐living diazotrophs and symbiotic diazotrophs are shown in red/purple and green, respectively. The purple square represents the PAS domain, the purple triangle represents the PAC domain, the green square represents the HisKA domain, the green triangle represents the HATPase domain, the blue bar represents the transmembrane structure, the purple pentagon represents the REC domain, and the yellow hexagon represents the HTH domain. PAS domain, Per‐Arnt‐Sim domain; PAC domain, PAS‐associated C‐terminal domain; HisKA domain, histidine kinase A domain; PHYLIP, phylogeny inference package; REC domain, receiver domain; HTH domain, helix–turn–helix domain.

To examine the distribution of the FixLJ two‐component system in diazotrophs, a phylogenetic tree was generated using the phylogeny inference package (PHYLIP) tree file obtained from the common taxonomy tree in the NCBI database. In addition to symbiotic diazotrophs, the *fixL* and *fixJ* genes encoding the FixLJ two‐component system were also identified in free‐living diazotrophs such as *R. palustris* (Figure 1A). To examine the genetic divergence of the FixLJ system, a functional genome clustering analysis was performed with seven symbiotic diazotrophs (*B. japonicum*, *Rhizobium gallicum*, *S. meliloti*, *A. caulinodans*, *Mesorhizobium japonicum*, *Rhizobium leguminosarum,* and *Rhizobium etli*) and nine additional purple non‐sulfur bacteria (PNSB) (*Rhodobacter capsulatus*, *Cereibacter sphaeroides*, *Rubrivivax gelatinosus*, *Rhodospirillum rubrum*, *Rhodomicrobium vannielii*, *Rhodopila* sp., *Blastochloris viridis*, *Rhodovulum sulfidophilum,* and *Pararhodospirillum photometricum*). No evolutionary gap of FixLJ could be identified between the free‐living diazotrophs and the symbiotic diazotrophs. Compared with the other PNSB, *R. palustris* showed a closer relationship with *B. japonicum* (Figure 1B). It retained both the FixLJ system of symbiotic diazotrophs and the photosynthesis system of PNSB. In addition, these *fixLJ*‐containing diazotrophs are widely distributed in nature, suggesting that the FixLJ system may play an important role in modulating the global nitrogen fixation. In addition, the FixL and FixJ from *R. palustris* showed almost the same protein domain architectures as that from *B. japonicum*. Both FixL have two sensory PAS domains, each with a PAC motif to facilitate its folding, and a histidine kinase (HK) module at the C‐terminus. As a typical transcriptional regulator, FixJ has a receiver (REC) domain at the N‐terminus and a helix–turn–helix (HTH) domain at the C‐terminus (Figure 1C). These data suggest that FixLJ regulates nitrogenase expression in response to oxygen levels not only in symbiotic diazotrophs but also in free‐living diazotrophs *R. palustris*. Comparative genome analysis revealed significant differences between free‐living *R. palustris* and symbiotic *B. japonicum* in substrate transport and signal transduction (Figure S1; Dataset S1).

### Deletion of *fixLJ* derepresses the nitrogen fixation under oxic conditions

**Figure 2:**
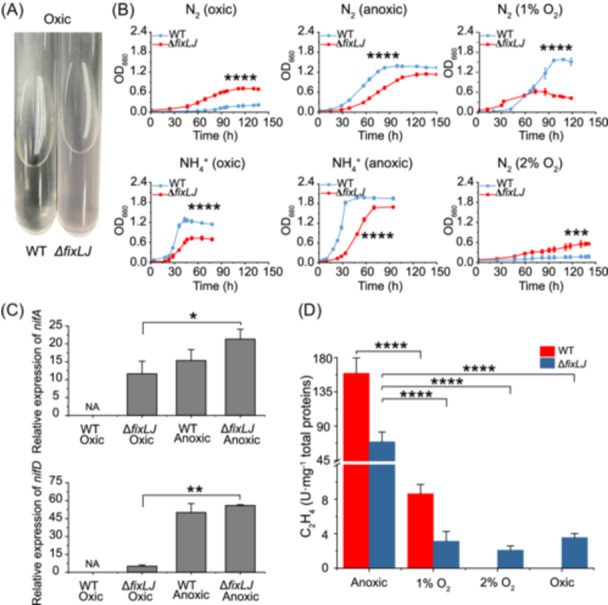
The deletion of *fixLJ* enables *Rhodopseudomonas palustris* to fix nitrogen under oxic conditions. (A) Diazotrophic growths of the wild‐type *R. palustris* CGA009 (WT) and its Δ*fixLJ* mutant CGA3015 (Δ*fixLJ*) under oxic conditions. (B) Growth curves of WT and Δ*fixLJ* under different oxygen conditions (oxic, anoxic, 1% O_2_, or 2% O_2_) with either NH_4_^+^ or N_2_ as the nitrogen source. *R. palustris* was grown in sealed culture tubes under illumination for anoxic growth, its oxic growth was carried out in an incubator shaker, and its micro‐oxic growth (1% O_2_ or 2% O_2_) was performed in an oxygen control glovebox. (C) Expression levels of *nifA* and *nifD* genes in WT and Δ*fixLJ* grown oxically or anoxically in nitrogen‐fixing medium. The transcript levels of the *nifA* and *nifD* genes were normalized to that of the *rpoD* gene. (D) Acetylene reduction activities of mid‐logarithmic phase of WT and Δ*fixLJ* cultures grown with N_2_ in the presence of different concentrations of oxygen (oxic, anoxic, 1% O_2_, or 2% O_2_). These data are the average of three independent experiments, and the error bars represent the SD. NA, not available. *p < 0.05; **p < 0.01; ***p < 0.001; ****p < 0.0001. *p* value was calculated using two‐way ANOVA (B) or one‐way ANOVA (C, D). ANOVA, analysis of variance.

The FixLJ two‐component system activates the transcription of *nifA* in the symbiotic diazotroph *S. meliloti* ^16^. To examine whether the FixLJ system plays a role in nitrogen fixation in the free‐living diazotroph *R. palustris*, we created an in‐frame deletion mutant of *fixLJ* (Δ*fixLJ*, strain CGA3015) and measured its diazotrophic growth under anoxic and oxic conditions. *R. palustris* was grown under illumination for anoxic growth according to a previously published method^17^, while its oxic or micro‐oxic growth was carried out with an incubator shaker, where *R. palustris* was cultured in 50‐ml culture tubes containing 10 ml of media at an agitation speed of 200 rpm. Both the wild‐type *R. palustris* CGA009 and the Δ*fixLJ* mutant were capable of diazotrophic growth under anoxic conditions. The wild‐type *R. palustris* CGA009 failed to grow in nitrogen‐fixing medium (NFM) with N_2_ as the nitrogen source under oxic conditions. However, we were surprised to find that the *R. palustris* Δ*fixLJ* mutant could grow diazotrophically under the same conditions (Figure 2A,B). We also examined their diazotrophic growth under micro‐oxic conditions (Figure 2B). Under a 1% oxygen condition, the wild‐type *R. palustris* grew better than the Δ*fixLJ* mutant. However, growth of the wild‐type *R. palustris* ceased when oxygen levels increased to 2%. These observations demonstrate that oxygen concentrations exceeding 2% can inhibit the diazotrophic growth of *R. palustris*. In contrast, the *R. palustris* Δ*fixLJ* mutant was still capable of growing under 1% and 2% oxygen conditions, albeit with reduced growth rates. These data suggest that the FixLJ system represses the expression of nitrogenase in the presence of oxygen.

To test this, we measured the expression levels of *nifA* and *nifD*, which encode the transcriptional activator and the α‐subunit of molybdenum (Mo) nitrogenase, respectively. Real‐time quantitative reverse transcription PCR (qRT‐PCR) showed that both *nifA* and *nifD* were expressed in the *R. palustris* Δ*fixLJ* mutant grown under oxic nitrogen‐fixing conditions. However, the expression level of *nifD* was 11‐fold lower than that in *R. palustris* grown under anoxic nitrogen‐fixing conditions (Figure 2C), suggesting that, as has been found in other bacteria^18^, NifA may not activate the expression of nitrogenase effectively when oxygen is present. Moreover, the *R. palustris* Δ*fixLJ* mutants showed acetylene reduction activities when cultured at various oxygen concentrations (ambient air, 1% oxygen, and 2% oxygen), albeit at relatively low levels compared to its performance under anoxic conditions (Figure 2D). In contrast, wild‐type *R. palustris* only showed acetylene reduction activity under anoxic and 1% oxygen conditions. These data would also explain the slow diazotrophic growth of the *R. palustris* Δ*fixLJ* mutant under oxic conditions.

### FixJ binds to the promoter region of *nifHDK*

**Figure 3:**
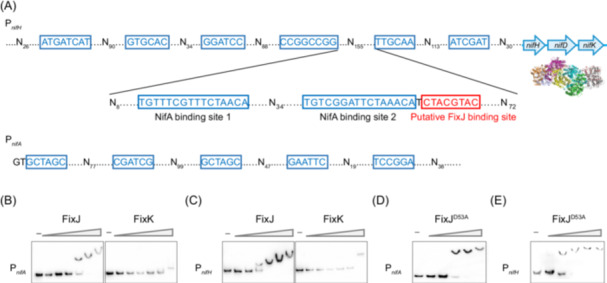
Unphosphorylated FixJ binds to the promoters of both *nifA* and *nifH*. (A) Putative FixJ binding site and palindromic sequences within P_*nifH*_ and P_*nifA*_ ^19^. (B–E) Measurement of the binding affinities of FixJ, FixK, and FixJ^D53A^ to P_*nifA*_ or P_*nifH*_ by electrophoretic mobility shift assays (EMSAs). An aliquot containing 5 nM P_*nifA*_ or P_*nifH*_ promoter was incubated with varied amounts of FixJ, FixK (0, 6.25, 12.5, 25, 50, 100, and 200 nM), or FixJ^D53A^ (0, 6.25, 25, 50, 100, and 200 nM), all of which were expressed and purified from *Escherichia coli*. N, any of the four nucleotide bases (A, T, C, or G) at the end of the legend. Blue box, palindromic sequences homologous to the FixJ binding site.

A FixJ DNA binding motif has been described and is located downstream of two NifA binding sites in the *R. palustris nifHDK* promotor region^19^ (Figure 3A). However, the *R. palustris nifA* promoter (P_*nifA*_) does not have a recognizable FixJ binding site^20^, ^21^. The palindromic sequences homologous to the FixJ binding site were identified in both P_*nifA*_ and P_*nifH*_. To test whether FixJ binds to P_*nifH*_ and P_*nifA*_, electrophoretic mobility shift assays (EMSAs) were carried out using FixJ and FixK proteins expressed and purified from *Escherichia coli* cells that were grown under oxic conditions. Our results show that FixJ binds to both P_*nifA*_ and P_*nifH*_, but with a higher binding affinity to P_*nifH*_. In contrast, FixK has, at best, a very low binding affinity to P_*nifA*_ and P_*nifH*_ (Figures 3B,C and S2). An increased shift of the FixJ–DNA complex was observed in the EMSA assays (Figure 3B,C), which may result from weak FixJ binding to these palindromic sequences. Since FixJ is not expected to undergo phosphorylation under oxic conditions^3^, the data presented above suggest that unphosphorylated FixJ represses nitrogenase synthesis. To confirm that the unphosphorylated FixJ binds to P_*nifA*_ and P_*nifH*_, we created a FixJ variant FixJ^D53A^ containing one amino acid substitution that disabled the phosphorylation reaction of FixJ^1^. It was found that the D53A amino acid substitution of FixJ (FixJ^D53A^) still showed binding affinities to P_*nifA*_ and P_*nifH*_ that were almost identical to that of the wild‐type FixJ (Figure 3D,E). These data suggest that unphosphorylated FixJ, or FixJ that has never been phosphorylated, directly regulates the expression of Mo nitrogenase.

### FixJ represses nitrogenase expression in response to oxygen

**Figure 4:**
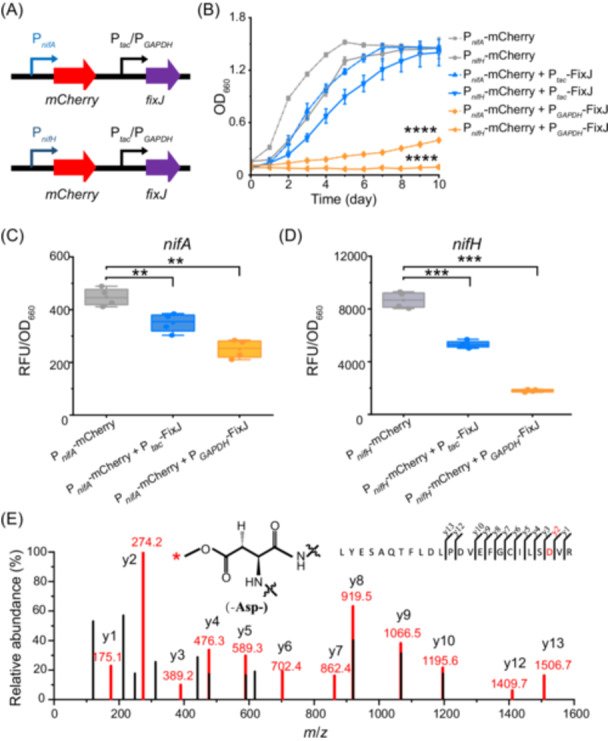
FixJ strongly inhibits the expression of the *nifHDK* gene cluster that encodes molybdenum (Mo) nitrogenase. (A) Red fluorescent protein (RFP) reporter systems that are used to measure the transcriptional activities of FixJ on *nifA* and *nifH*, respectively. (B) Diazotrophic growth of *R. palustris* overexpressing *fixJ* under anoxic conditions. Cell growths were monitored with time at OD_660_. Data are representative of three independent measurements, and the error bars represent the SD. The *p* value was calculated using two‐way ANOVA. (C, D) Transcriptional regulation of *nifA* (C) or *nifH* (D) by FixJ. Statistics 17.0 software (SPSS) was used to perform statistical analysis. The Duncan method was used to calculate differences between multiple groups and were analyzed by one‐way ANOVA. RFU, relative fluorescence unit. Data are averages of four independent measurements, and the maximum, median, and minimum values are shown. **p < 0.01; ***p < 0.001; ****p < 0.0001. (E) Nano liquid chromatography‐tandem mass spectrometry (nano‐LC‐MS/MS) analysis showing that the Asp^53^ residue of the FixJ regulator was in an unphosphorylated state under oxic conditions.

To confirm that FixJ represses the expression of nitrogenase, a red fluorescent protein (RFP) reporter system was constructed and the expression levels of *nifA* and *nifH* were determined (Figure 4A)^22^. *R. palustris* strains overexpressing FixJ controlled by a strong promoter P_*GAPDH*_ grew poorly under anoxic nitrogen‐fixing conditions (Figure 4B). However, expression of FixJ under the control of a weak promoter P_*tac*_ (about 50% of the strength of P_*GAPDH*_) had less influence on the diazotrophic growth of *R. palustris* and the gene expression of *nifA* and *nifH* (Figure 4). Against this background of poor growth, the transcription levels of *nifA* and *nifH* decreased with increased expression levels of FixJ (Figure 4C,D). The FixJ interfered with *nifA* expression slightly, suggesting that FixJ was probably a weak inhibitory regulator of *nifA* transcription. However, the transcription level of *nifH* was dramatically lower when FixJ was overexpressed with the strong promoter P_*GAPDH*_. In addition, nano liquid chromatography‐tandem mass spectrometry (nano‐LC‐MS/MS) analysis revealed no detectable phosphorylation of the Asp^53^ residue of FixJ under oxic conditions (Figure 4E), which is consistent with the fact that FixL is inactive with a heme Fe(II) − O_2_ complex in the PAS domain^3^. Taken together, our data show that unphosphorylated FixJ functions to repress transcription of nitrogenase genes.

**Figure 5:**
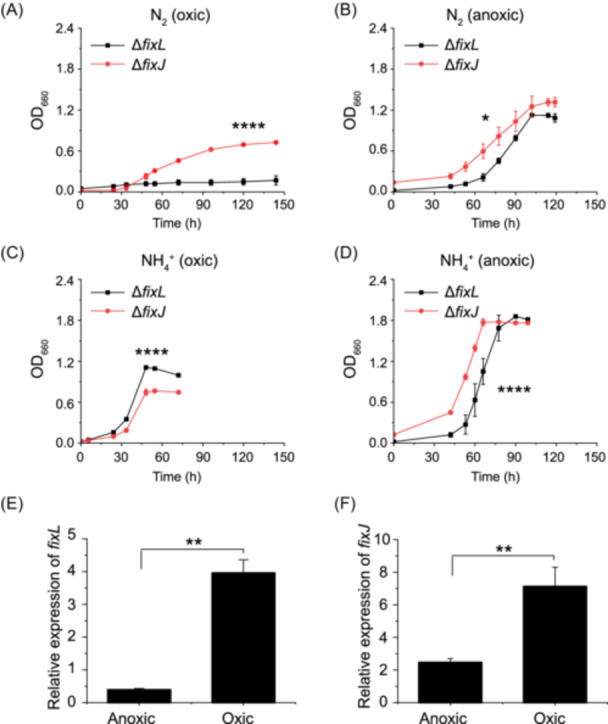
FixJ is responsible for the depression of nitrogenase expression under oxic conditions. (A–D) Growth curves of *R. palustris* Δ*fixL* and Δ*fixJ* mutants under oxic and anoxic conditions with either NH_4_^+^ or N_2_ as the nitrogen source. For anoxic growth, *R. palustris* was grown in sealed culture tubes under illumination, while oxic growth was achieved by shaking at 200 rpm in an incubator shaker. The *p* values were calculated using two‐way ANOVA. (E, F) Expression levels of *fixL* and *fixJ* under anoxic or oxic conditions. Real‐time quantitative reverse transcription (qRT‐PCR) was used to determine the gene expression of the wild‐type *R. palustris* CGA009 grown on a defined mineral medium supplemented with NH_4_^+^. The transcript levels of the *fixL* and *fixJ* genes were normalized to that of the *rpoD* gene. The Duncan method was used to calculate differences between multiple groups and were analyzed by one‐way ANOVA. Data are representative of three independent measurements, and the error bars represent the SD. *p < 0.05; **p < 0.01; ****p < 0.0001.

Given that unphosphorylated FixJ is required for nitrogenase regulation in response to oxygen, the FixL component should be disposable in this process. Therefore, we constructed the *R. palustris* Δ*fixJ* single mutant to confirm that the deletion of a single *fixJ* gene can depress the nitrogenase expression under oxic conditions. As shown in Figure 5A, the Δ*fixJ* mutant was able to grow diazotrophically under oxic conditions, showing the similar growth phenotype as the Δ*fixLJ* double mutant (Figure 2B). In contrast, the *fixL* mutant (Δ*fixL*) grown under oxic conditions with N_2_ showed a growth phenotype similar to that of the wild type (Figure 2B), as the unphosphorylated FixJ retained in this mutant was still able to repress nitrogenase expression (Figure 5A). Similar to the Δ*fixLJ* mutant (Figure 2B), both Δ*fixL* and Δ*fixJ* mutants were able to grow diazotrophically under anoxic conditions, which is attributed to the low expression level of *fixLJ* (Figure 5B). Under nitrogen‐sufficient conditions, the deletion of *fixL* or *fixJ* has little effect on cell growth (Figure 5C,D).

To determine if the transcription of *fixLJ* genes is stimulated by oxygen, qRT‐PCR was used to quantify the expression levels of *fixLJ* genes under oxic and anoxic conditions. The expression of *fixL* and *fixJ* significantly increased when the wild‐type *R. palustris* CGA009 grown anoxically was shifted to oxic growth under nitrogen‐excess conditions (Figure 5E,F). Under oxic conditions, the increased amount of FixJ would be expected to reinforce nitrogenase repression, because of which the wild‐type *R. palustris* strain is unable to grow diazotrophically.

### FixLJ is involved in the regulation of energy and nitrogen metabolism

**Figure 6:**
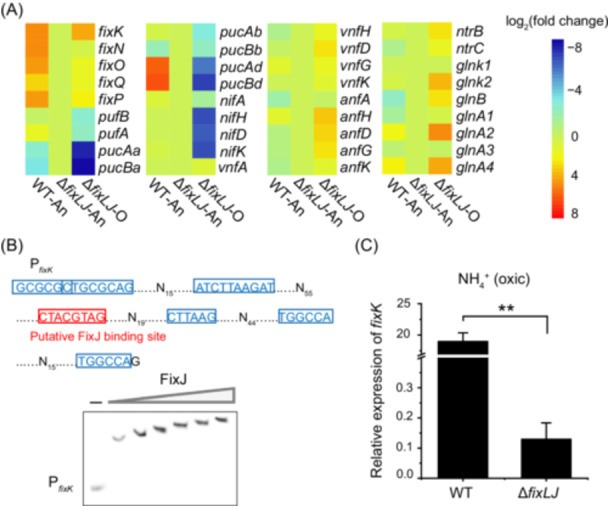
The oxygen‐sensing FixLJ system stimulates the expression of global transcriptional regulator FixK that further regulates the genes involved in photosynthesis and oxidative phosphorylation. (A) Heat map illustrating the genes of great importance to the diazotrophic growth of the *R. palustris ΔfixLJ* mutant differentially expressed under oxic/anoxic conditions in nitrogen‐fixing medium. *R. palustris* was grown in sealed culture tubes under illumination for anoxic growth, whereas oxic growth was carried out by shaking at 200 rpm in an incubator shaker. An, anoxic; O, oxic. (B) Measurement of the binding affinities of FixJ to P_*fixK*_ by EMSA. FixJ could strongly bind to the promoter of *fixK*. An aliquot containing 5 nM P_*fixK*_ promoter was incubated with varied amounts of FixJ (0, 6.25, 12.5, 25, 50, 100, and 200 nM), which was expressed and purified from *E. coli*. (C) Expression level of *fixK* under oxic conditions. qRT‐PCR was used to determine the gene expression of WT and Δ*fixLJ* grown on a defined mineral medium supplemented with NH_4_^+^. The oxic growth of *R. palustris* strains was achieved by shaking at 200 rpm in an incubator shaker. The transcript level of the *fixK* gene was normalized to that of the *rpoD* gene. Data are representative of three independent measurements, and the error bars represent the SD. **p < 0.01. The *p* values were calculated using one‐way ANOVA.

To gain more insights into the characteristics of oxic nitrogen fixation, we compared the transcriptomes of the wild type grown anoxically and the Δ*fixLJ* mutant grown anoxically/oxically in nitrogen‐fixing medium (SRA database: PRJNA1091417). The global regulator gene *fixK*, whose protein product (FixK) plays a role in the energy metabolism of *R. palustris* ^11^, was downregulated in the Δ*fixLJ* mutant grown anoxically (Figure 6A; Dataset S2). To confirm that the expression of the *fixK* gene is activated by the FixLJ system, we determined the binding affinity of FixJ to the promoter of the *fixK* gene (P_*fixK*_) by EMSA, and quantified the expression of *fixK* in the presence or absence of *fixLJ*. FixJ showed strong binding affinity to P_*fixK*_ (Figure 6B), whose promoter harbors the consensus motif “GTACGTAG,” and the transcription of *fixK* drastically decreased upon deletion of *fixLJ* in *R. palustris* grown under oxic conditions (Figure 6C). Thus, the expression of the global transcriptional regulator FixK is stimulated by FixLJ in response to the environmental oxygen status. Other important gene clusters expressed at lower levels included *cbb*_3_‐type terminal oxidase genes (*fixNOQP*) and light‐harvesting complex genes (*puf* and *puc*) (Figure 6A), which are all positively regulated by FixK.

With regard to the nitrogenase genes under oxic conditions in the Δ*fixLJ* mutant, the downregulated expression and impaired activity of NifA likely lead to the observed decreased expression of Mo nitrogenase (*nifHDK*). In contrast to Mo nitrogenase, alternative nitrogenase‐related genes (*vnfA*, *vnfDGK*, *anfA*, and *anfDGK*), along with *anf3*, which are not normally expressed when Mo nitrogenase is available, were upregulated. Additionally, *ntrBC*, *glnK*, *glnB,* and *glnA* genes, which are involved in nitrogen regulation and nitrogen assimilation, were also upregulated when the Δ*fixLJ* mutant was grown oxically in nitrogen‐fixing medium (Figure 6A). These data indicate that limited fixed nitrogen caused by the low expression of Mo nitrogenase stimulates *R. palustris* cells to increase the expression of genes that are important for fixed nitrogen acquisition.

## DISCUSSION

The FixLJ–FixK regulatory system is widespread in α‐proteobacteria, where it regulates a variety of functions in response to low oxygen. It has been most studied in *S. meliloti*, *B. japonicum,* and *Azospirllum caulinodans* in the context of micro‐oxic conditions similar to those that exist in the nitrogen‐fixing symbiotic root nodules that *S. meliloti* and *B. japonicum* form. In *A. caulinodans*, FixLJ further activates the expression of *fixK* and the FixK protein stimulates the expression of NifA^13^, in *S. meliloti*, the expression of NifA is activated by FixJ‐P^23^, and in *B. japonicum*, FixK indirectly modulates the activity of NifA by controlling the synthesis of the alternative sigma factor RpoN (σ^54^)^24^, ^25^. The disruption of *fixL* or *fixJ* in *B. japonicum* causes a 90% decrease in its performance of nitrogen fixation^26^. In this study, we found that expression of the *fixLJ* two‐component regulatory system in *R. palustris* was stimulated by oxygen (Figure 5); moreover, unphosphorylated FixJ repressed the expression of nitrogenase genes by directly binding to the promoter of *nifHDK*. We also showed that the deletion of *fixLJ* genes allowed *R. palustris* to grow diazotrophically under oxic conditions. However, growth was slow and cell yields were lower than expected (Figure 2), probably because of the extreme sensitivity of nitrogenase to oxygen. In any case, the repression of nitrogenase expression by FixJ in the presence of oxygen appears to be a new activity for this two‐component regulatory system.

The obligately oxic diazotrophs such as *Azotobacter vinelandii* have developed distinct protective mechanisms to shield nitrogenase from the deleterious effects of oxygen. One of the protective strategies is respiratory protection, which utilizes the robust respiratory activity facilitated by numerous terminal oxidases to maintain an anoxic environment for nitrogenase, though there is some debate over its importance^27^. Another strategy is known as conformational protection, which takes advantage of a flavocytochrome‐encoded by *anf3* function under oxic condition^28^. *R. palustris* also encodes a homolog of the Anf3 (RPA1432), which can protect nitrogenase from oxygen when *R. palustris* is exposed to small amounts of oxygen. The expression of the *anf3* gene was upregulated in response to oxygen, showing a 2.2‐/6.9‐fold increase compared to the culture of the Δ*fixLJ* mutant or wild type grown under anoxic conditions (Dataset S2). As oxygen concentrations increase, Anf3 may protect Fe‐only nitrogenase from oxygen damage in the slow‐growing *R. palustris* cells for living. In addition, hopanoids potentially play a role in nitrogenase protection, given that they can act as an oxygen diffusion barrier by lowering the permeability to extracellular oxygen^29^, ^30^. However, the expression level of the *sqhC* gene involved in hopanoid production was relatively low, suggesting its minimal contribution to nitrogenase protection. To avoid synthesizing too much useless inactive nitrogenase, *R. palustris* prefers shutting down nitrogenase expression by FixJ during oxic growth in nature (Figures 3 and 4).

Given that nitrogen fixation is an energy‐intensive process that needs at least 16 ATP to fix one N_2_, energy metabolism is of great importance to the diazotrophic growth. In *R. palustris,* oxygen induces the expression of FixLJ (Figure 5), which further activates the global transcriptional regulator FixK. Transcriptome analyses of the *R. palustris* Δ*fixK* mutant have shown that FixK positively regulates a wide range of genes such as heme and bacteriochlorophyll biosynthesis, *cbb*_3_ oxidase, and NADH dehydrogenase genes^11^. Therefore, the deletion of *fixLJ* caused the downregulation of genes involved in photosynthesis (*puc* and *puf* genes*)* and oxidative phosphorylation (*fixNOQP*) by downregulating the *fixK* gene that codes for FixK (Figure 6). In addition, synthesis of the photosynthetic apparatus is blocked under oxic conditions, causing a decrease in pigmentation and photosystem^31^, ^32^. We also note that the *R. palustris* Δ*fixLJ* mutant grew more slowly than the wild type under anoxic conditions (Figure 2B). This is likely because FixLJ activates the expression of FixK under anoxic conditions and FixK in turn activates the expression of genes required for photophosphorylation^11^. The reduced supply of ATP from either photophosphorylation or oxidative phosphorylation may contribute to the impaired growth of the *R. palustris* Δ*fixLJ* mutant under oxic conditions.

Compared with the wild‐type *R. palustris* CGA009 grown anoxically with molybdenum, a condition that only induces the expression of Mo nitrogenase^33^, the *R. palustris* Δ*fixLJ* mutant grown oxically with molybdenum significantly increased the expression of alternative nitrogenases (*vnfHDGK* and *anfHDGK*) and Anf3 which is homologous to *A. vinelandii* flavocytochrome working toward oxygen consumption^28^, in contrast to the decreased expression of Mo nitrogenase (*nifHDK*) (Figure 6). The inability to fix sufficient nitrogen probably stimulated the expression of alternative nitrogenase protected by Anf3 to support the diazotrophic growth of the *R. palustris* Δ*fixLJ* mutant under oxic conditions (Figure 6A). In addition, in response to intracellular nitrogen limitation, the expression levels of nitrogen regulatory proteins such as two‐component regulators NtrB‐NtrC, P_II_ proteins and glutamine synthase were also upregulated under oxic conditions. In *R. palustris*, nitrogen fixation was activated by the NtrBC‐P_II_ cascade via targeting NifA^18^. The significantly increased expression of NtrBC and P_II_ proteins could be used to compensate the partly lost activity of NifA caused by oxygen.

**Figure 7:**
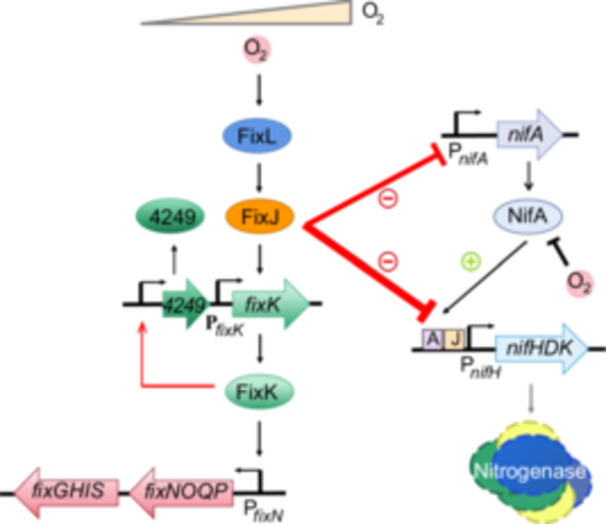
Model for the regulation of nitrogenase in response to oxygen availability in *R. palustris*. The green plus in a circle represents activation, while the red minus in a circle represents inhibition. Box A, NifA binding site; Box J, FixJ binding site; and *4249*, the *rpa4249* gene encodes the protein RPA4249 (4249), which is homologous to the regulatory protein FixT.

Based on the data that we have obtained in *R. palustris*, a regulatory model of nitrogenase expression in response to oxygen was proposed for the photosynthetic diazotroph (Figure 7). When exposed to oxygen, *R. palustris* activates the expression of the FixLJ system. The activated FixJ further stimulates the expression of FixK, which can induce the oxidative phosphorylation process to provide *R. palustris* cells with more energy. In addition, FixJ strongly represses the expression of Mo nitrogenase by binding to the promoter of *nifHDK*, and in the meantime, the transcriptional activator of Mo nitrogenase (NifA) is inactivated by oxygen (Figure 7). *R. palustris* finally shuts down the nitrogenase expression to avoid wasting energy, since it seems to lack a powerful functional oxygen‐protecting mechanism for nitrogenase^28^, ^34^.

Altogether, this study uncovers a novel regulatory role of the two‐component system FixLJ in the free‐living phototrophic diazotroph *R. palustris*, where it represses nitrogenase expression in response to oxygen. We demonstrate that unphosphorylated FixJ binds directly to the promoter of the nitrogenase gene cluster, thereby shutting down nitrogenase synthesis under oxic conditions. This mechanism prevents the accumulation of oxygen‐damaged nitrogenase and also prevents unnecessary energy expenditure. Our findings highlight a previously unrecognized strategy by which free‐living diazotrophs regulate nitrogen fixation in fluctuating oxygen environments, thereby deepening our understanding of microbial adaptation to environmental challenges and providing a theoretical foundation for the development of synthetic nitrogen fixation technologies and sustainable agriculture.

## MATERIALS AND METHODS

### Bacterial strains and growth conditions

For genetic manipulation, *R. palustris* was grown oxically at 30°C using photosynthetic medium (PM) agar supplemented with 10 mM sodium succinate^17^. *E. coli* S17‐1 was grown on LB medium at 37°C. For diazotrophic growth, *R. palustris* strains were grown in light at 30°C anoxically or micro‐oxically or oxically in NFM, which is the PM but lacks ammonium sulfate^35^. For non‐diazotrophic growth, *R. palustris* strains were grown anoxically or oxically in 10 ml of PM medium, maintaining the same light and temperature conditions as those used for diazotrophic growth. Final concentrations of 20 mM sodium acetate and 10 μM sodium molybdate (Na_2_MoO_4_) were added to the medium. Anoxic environments were maintained by filling N_2_ gas in the headspace of the sealed culture tubes, micro‐oxic assays were performed in an oxygen control glove box (Coy Laboratory Products), and oxic environments were created by shaking 10 ml of medium in culture tubes (1.8 cm × 20 cm) made of borosilicate glass capped with sponge silicone plugs at 200 rpm only in the lab environment. For micro‐oxic conditions, autoclaved culture tubes filled with 10 ml medium were rapidly transferred to an oxygen‐controlled glovebox and equilibrated for 1 week prior to use. Illumination was also provided for micro‐oxic and oxic growths of *R. palustris* using a 40 W LED bulb positioned 20–30 cm from the cultures. When appropriate, *E. coli* and *R. palustris* cells were grown with gentamicin at 20 and 100 μg/ml, respectively.

### Genetic manipulation of *R. palustris*

All strains and plasmids used in this work are shown in Dataset S3. *R. palustris* Δ*fixLJ* (CGA3015), Δ*fixL* (CGA3045), and Δ*fixJ* (CGA3046) mutants were prepared as follows: PCR was used to amplify about 1 kb of DNA upstream of the start codon for *fixL* and about 1 kb of DNA downstream of the stop codon for *fixJ*. These DNA fragments were then inserted into the *Pst*I‐digested pJQ‐200SK vector using a T5 exonuclease‐dependent assembly (TEDA) method^36^. In‐frame deletions of *fixL* (1516 bp; the start codon was reserved and the stop codon was added upstream of the *fixJ* gene) or *fixJ* (600 bp; the start codon and the stop codon were reserved) were created by PCR using the Q5 DNA polymerase to amplify about 1 kb of DNA upstream of the start codon and about 1 kb of DNA downstream of the stop codon for *fixL* or *fixJ*. These DNA fragments were then inserted into *Bam*HI‐digested pJQ200SK using the above‐mentioned TEDA method^36^. Plasmids pJQ‐Δ*fixLJ*, pJQ‐Δ*fixL*, and pJQ‐Δ*fixJ* were first transformed into *E. coli* S17‐1 and then mobilized into *R. palustris* CGA009 by conjugation, and a *fixLJ*, *fixL, or fixJ* deletion mutant was generated using a screening strategy as previously described^37^. CGA3015, CGA3045, and CGA3046 were finally obtained, respectively.

For the construction of RFP reporter systems *in vivo*, the *mCherry* gene fused with the promoter of *nifA* or *nifH* was incorporated into the *Eco*RI‐digested pBBR1MCS‐6, yielding plasmids pBBR1MCS‐P_*nifA*_‐RFP and pBBR1MCS‐P_*nifH*_‐RFP, respectively. DNA fragments containing *fixJ* controlled by a strong promoter P_*GAPDH*_ or a weak promoter P_*tac*_ were inserted into *Hin*dIII‐digested pBBR1MCS‐P_*nifA*_‐RFP and pBBR1MCS‐P_*nifH*_‐RFP, respectively^22^. The obtained plasmids were mobilized into *R. plaustris* CGA009 by conjugation, generating a series of *R. palustris* strains (CGA009‐1 to CGA009‐8). The DNA fragment containing *fixLJ* genes under the control of P_*GAPDH*_ was incorporated into *Eco*RI‐digested pBBR1MCS‐6. The obtained plasmid pBBR1MCS‐P_*GAPDH*_‐*fixLJ* was then mobilized into *R. plaustris* CGA3015, generating *R. palustris* CGA3015‐1.

### Acetylene reduction assays

Nitrogenase activity was measured by monitoring acetylene (C_2_H_2_) reduction to ethylene (C_2_H_4_). 4 ml of culture withdrawn from 10‐ml cultures grown anoxically in NFM to the exponential phase was introduced into a 25‐ml sealed tube filled with argon and 2% C_2_H_2_. In regard to the culture grown oxically or micro‐oxically (1% or 2% oxygen) in NFM, cells harvested from a 4‐ml aliquot of a 10‐ml exponential‐phase culture were washed three times with anoxic NFM, and then transferred to a 25‐ml sealed culture tube that was filled with argon. After incubation with light at 30°C for 4 h, a final concentration of 2% acetylene was added to the sealed tube to initiate acetylene reduction. The C_2_H_4_ produced in the headspace was quantified as described previously^33^.

### qRT‐PCR analysis

*R. palustris* cells grown to the logarithmic phase were first harvested. Then, the total RNA was isolated from samples using the RNA simple Total RNA Kit (TIANGEN Biotech) according to the instructions of the manufacturer. Next, 2 μg of total RNA was used to prepare cDNA using the FastKing‐RT SuperMix Kit (TIANGEN Biotech). During cDNA preparation, the reaction mixture was incubated at 42°C for 15 min to complete reverse transcription and digest the genomic DNA. The primers used for qRT‐PCR are listed in Table S1. The qRT‐PCR reactions were performed with the LightCycler 480 II Real‐Time PCR System (Roche Diagnostics) using the SuperReal PreMix Plus Kit (TIANGEN Biotech). Transcriptional level of the housekeeping gene *rpoD* was used as an internal standard^38^.

### Transcriptome analysis

Transcriptome sequencing was carried out by Shanghai Majorbio Bio‐pharm Technology Co. Ltd. Total RNA quality was examined by the 2100 Bioanalyser (Agilent). The TruSeq^TM^ Stranded Total RNA Library Prep Kit from Illumina was used to prepare the RNA‐seq transcriptome library. The paired‐end RNA‐seq sequencing library was then sequenced with Illumina HiSeq×10 (2 × 150 bp read length). A Perl program was used to obtain clean reads. A free online platform from Majorbio was used to analyze the data. The basic functional annotation was carried out according to the reference genome (BX571963.1). The gene expression levels were calculated using the TPM (Transcripts Per Million reads) method. The “DESeq. 2” R package was used to identify the differentially expressed genes with the parameters *p*‐adjust < 0.05 and |log_2_FC| ≥ 1. Heatmaps of differentially expressed genes were drawn using the R package “pheatmap.”

### Identification of phosphorylation of the Asp residue of the FixJ protein

The plasmid pBBR1MCS6‐P_*GAPDH*_‐*fixLJ* (Table S1) was created by incorporating the P_*GAPDH*_‐*fixLJ* sequence into *Eco*RI/*Kpn*I‐digested pBBR1MCS6. The obtained pBBR1MCS‐P_*GAPDH*_‐*fixLJ* was mobilized into *R. palustris* Δ*fixLJ* by conjugation with the *E. coli* S17‐1 strain. The obtained *R. palustris* CGA3015‐1 was grown oxically, with its cell pellet harvested by centrifugation. Cell extracts from *R. palustris* CGA3015‐1 were prepared using a high‐pressure cell crusher before sodium dodecyl sulfate polyacrylamide gel electrophoresis (SDS‐PAGE) analysis. Proteins within the range of 20–30 kDa were selected for nano‐LC‐MS/MS analysis. MS/MS data were analyzed using the Mascot search engine (v.2.8.0, 2021; Matrix Science Ltd.) (Figure 4E). Carbamidomethylation on cysteine was specified as fixed modification, while phosphorylation on aspartate and oxidation on methionine were specified as variable modifications. The threshold values of the false discovery rate for modification sites and peptides were specified at 1%. The default values were used for other parameters in Mascot.

### Protein expression and purification

The plasmids pET28a::*fixJ* and pET28a::*fixK* were created by incorporating the *fixJ* and *fixK* coding sequences obtained by PCR amplification into the *Bam*HI‐digested pET28a, respectively, using a TEDA method^36^. Plasmid pET28a::*fixJ* ^*D53A*^ was generated by site‐directed mutation using primers D53A‐F and D53A‐R as templates. *E. coli* BL21(DE3) was used to overexpress the recombinant proteins. The harvested recombinant cells were first suspended in Buffer A (20 mM Tris‐HCl [pH 7.5], 20 mM imidazole, and 300 mM NaCl), and then lysed by sonication, followed by centrifugation at 10,000 rpm for 1 h at 4°C. The supernatant collected was loaded onto the HisTrap Excel column (Cytiva). The target protein was eluted with increasing proportions of Buffer B (20 mM Tris‐HCl [pH 7.5], 500 mM imidazole, and 300 mM NaCl) after washing with Buffer A. The purified protein was concentrated to 2 mM by ultrafiltration.

### Protein–DNA interactions

EMSA was used to identify the protein–DNA interactions. The promoters (P_*nifA*_, P_*nifH*_, and P_*fixK*_) were labeled with biotin‐11‐dUTP at their 5′‐ends using the EMSA Probe Biotin Labeling Kit (Beyotime Biotechnology). After incubation for 20 min at room temperature, the prepared proteins and DNA probes were loaded onto a 9% non‐denaturing polyacrylamide gel. Polyacrylamide gel electrophoresis was run in an ice‐cold Tris‐borate‐EDTA (TBE) buffer. The gel was subsequently printed onto a positively charged nylon membrane, followed by UV‐activated crosslinking for 5 min. Band visualization was carried out using an automated chemiluminescence image analyzer.

### Measurement of the fluorescence intensity

Measurement of the RFP fluorescence intensity was carried out based on a standard protocol described previously^22^. When *R. palustris* cells were grown to the exponential phase, they were harvested by centrifugation. The cell pellets were then suspended using 10 mM phosphate buffer (PBS), followed by pipetting 200 μl aliquots into the 96‐well black and clear microplates to read the absorption of relative fluorescence intensity and OD_660_ by the microplate reader (BioTek Synergy TMH4). The excitation and emission wavelengths were set at 587 and 610 nm, respectively.

### Bioinformatic analysis

The homologous protein sequences were aligned using the ClustalW program of MEGA 7 software to calculate the Poisson correction distance. Phylogenetic trees were created by MEGA 7 software using the neighbor‐joining algorithm. To test the reliability of trees, bootstrap tests were carried out with 1000 replicates. An online database SMART (Simple Modular Architecture Research Tool) was applied to analyze the protein domain architectures^39^. R package “KEGGREST” was used to retrieve all genes from KEGG pathways of *R. palustris* CGA009 and *B. japonicum* E109. The top 24 pathways ranked by differential gene counts were visualized as a Sankey diagram using OriginPro 2022 beta.

## AUTHOR CONTRIBUTIONS

**Lingwei Cui**: Data curation; formal analysis; investigation; writing—original draft; writing—review and editing. **Yan Zeng**: data curation; formal analysis; funding acquisition; investigation; methodology; writing—original draft; writing—review and editing. **Mengmei Wang**: Investigation; writing—review and editing. **Lu Huang**: Investigation; writing—review and editing. **Zheyi Wang**: Investigation; writing—review and editing. **Ying Liu**: Investigation; writing—review and editing. **Yanning Zheng**: Conceptualization; funding acquisition; project administration; supervision; writing—review and editing.

## ETHICS STATEMENT

This study did not involve animal experiments or include human samples.

## CONFLICT OF INTERESTS

The authors declare no conflict of interests.

## Supporting information


**Figure S1.** Sankey diagram of the top 24 KEGG pathways ranked by differential gene counts between *R. palustris* CGA009 and *B. japonicum* E109.
**Figure S2.** Measurement of the binding affinities of FixJ and FixK to no‐specific DNA (P_*anfA*_) by EMSA.

**Data Set S1.xlsx.** The top 24 KEGG pathways ranked by differential gene counts between *R. palustris* CGA009 and *B. japonicum* E109.

**Data Set S2.xlsx.** Transcriptomic data sets from the *R. palustris* Δ*fixLJ* mutant grown oxically and anoxically, respectively, and from the wild‐type *R. palustris* grown anoxically.

**Data Set S3.xlsx.** Plasmids and primers used in this study.

## Data Availability

Transcriptomic data generated in this study have been deposited in the NCBI Sequence Read Archive under the accession number PRJNA1091417.
