# Supplementary material for: The oxygen‐sensing FixLJ represses nitrogen fixation in Rhodopseudomonas palustris in response to oxygen
Source: mLife. 2026 Apr 9;5(3):312–24. doi: 10.1002/mlf2.70067 (PMC13327599; doi:10.1002/mlf2.70067)
Supplement: Supplementary file 1 — Figure S1. Sankey diagram of the top 24 KEGG pathways ranked by differential gene counts between R. palustris CGA009 and B. japonicum E109. Figure S2. Measurement of the binding affinities of FixJ and FixK to no‐specific DNA (P anfA ) by EMSA. [file MLF2-5-312-s001.pdf]

Supporting Information for

The oxygen-sensing FixLJ represses nitrogen fixation in *Rhodopseudomonas palustris* in response to oxygen

Lingwei Cui[1,2,†], Yan Zeng[1,†], Mengmei Wang[1,2], Lu Huang[1,2], Zheyi Wang[1,2], Ying Liu[1], Yanning Zheng[1,2,*]

[1] State Key Laboratory of Microbial Diversity and innovative Utilization, Institute of Microbiology, Chinese Academy of Sciences, Beijing 100101, China.

[2] College of Life Sciences, University of Chinese Academy of Sciences, Beijing 100049, China.

[†] These authors contributed equally.

[*] Correspondence: Yanning Zheng, zhengyn@im.ac.cn.

This PDF file incudes:

Figure S1

Figure S2

Figure S1

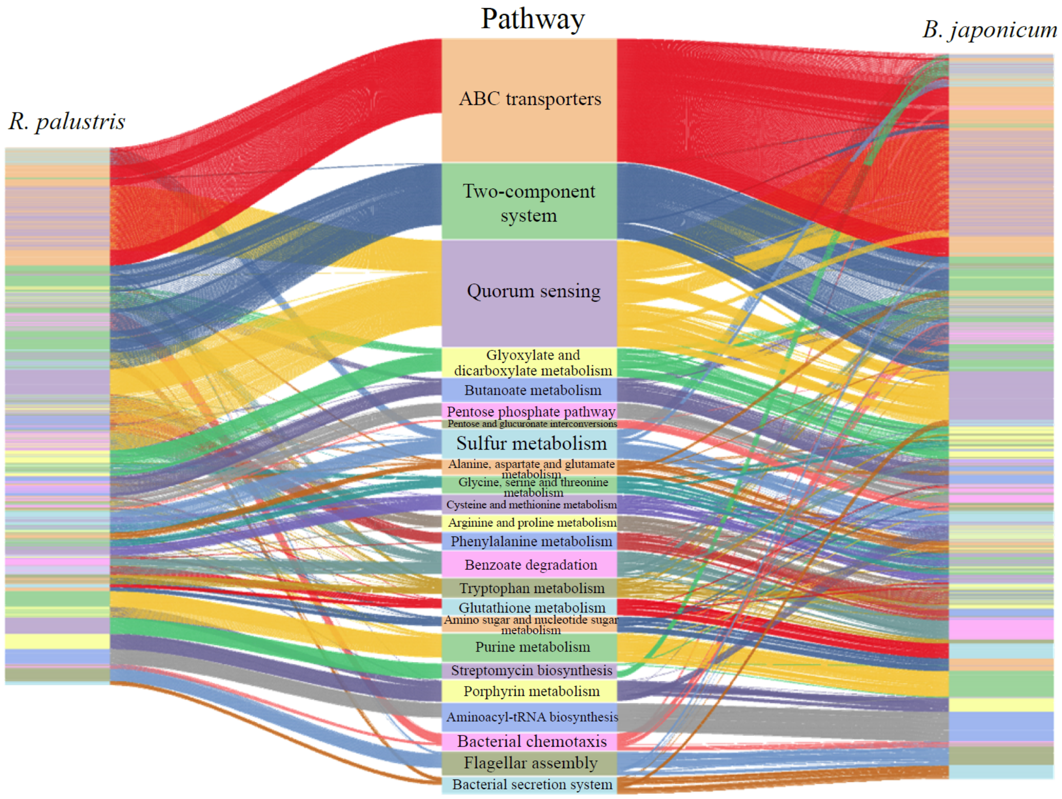

Figure S1. Sankey diagram of the top 24 KEGG pathways ranked by differential gene counts between *R. palustris* CGA009 and *B. japonicum* E109.

Figure S2

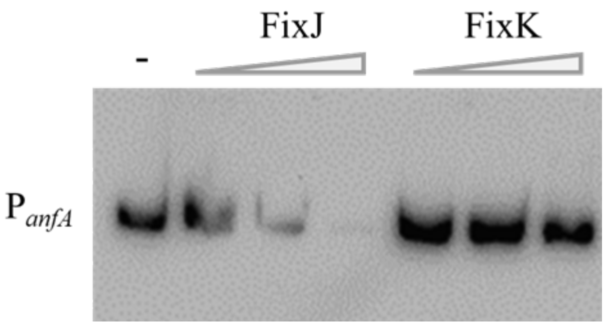

Figure S2. Measurement of the binding affinities of FixJ and FixK for no-specific DNA (P_{anfA}) by EMSA. No band-shift was observed in the EMSA assay, suggesting that FixJ binding to DNA is sequence-specific. P_{anfA} has no binding site of FixJ or FixK. An aliquot containing 5 nM P_{anfA} was incubated with varied amounts of FixJ or FixK (50, 100 and 200 nM).
